# Serum creatinine outperforms the creatinine to cystatin C ratio and creatinine muscle index as biomarkers in Chinese pediatric spinal muscular atrophy

**DOI:** 10.3389/fnhum.2026.1764456

**Published:** 2026-04-02

**Authors:** Zixi Huang, Yingshuang Peng, Ping Yuan, Siqi Hong, Li Jiang

**Affiliations:** Department of Neurology, Children’s Hospital of Chongqing Medical University, National Clinical Research Center for Children and Adolesents’ Health and Diseases, Ministry of Education Key Laboratory of Child Development and Disorders, Chongqing Key Laboratory of Child Neurodevelopment and Cognitive Disorders, Chongqing, China

**Keywords:** biomarker, creatinine, creatinine muscle index, creatinine to cystatin C ratio, spinal muscular atrophy

## Abstract

**Background:**

This study aimed to validate the utility of serum creatinine (Cr), creatinine-to-cystatin C ratio (CCR) and creatinine muscle index (CMI) as biomarkers in Chinese pediatric patients with spinal muscular atrophy (SMA).

**Methods:**

A retrospective cohort of 21 pediatric patients with 5q-spinal muscular atrophy (5q-SMA) was followed over 18-month after initiating nusinersen therapy. Serum biomarkers (creatinine, cystatin C, and albumin) and multidimensional motor function scores were assessed at six predefined time points. Associations between serum markers and motor function outcomes were analyzed statistically.

**Results:**

A robust and sustained correlation was identified between Cr levels and all motor function metrics—both at baseline and during follow-up (Spearman’s *ρ* ≥ 0.711, *p* < 0.001). This association remained statistically significant even after adjusting for confounders (age, SMA subtype, and albumin levels). In contrast, the baseline relationship between CCR and motor function was attenuated post-adjustment, suggesting confounding effects. CMI exhibited no significant associations in any analysis. Longitudinally, neither Cr nor CCR displayed marked changes over time, whereas CMI declined significantly at the V8 assessment (*p* = 0.016).

**Conclusion:**

Routinely measured Cr is a more reliable dynamic biomarker than CCR and CMI for assessing therapeutic response in pediatric SMA patients undergoing nusinersen treatment.

## Highlights

- This is the first study to systematically evaluate serum creatinine (Cr), creatinine-to-cystatin C ratio (CCR), and creatinine muscle index (CMI) as monitoring biomarkers in Chinese children with SMA. Our results demonstrate Cr’s unique clinical relevance in this understudied population.
- We establish Cr as a cost-effective and accessible alternative to composite indices (CCR, CMI) for tracking SMA progression. Its simplicity is particularly valuable for resource-limited settings, offering potential to refine global SMA management guidelines.

## Introduction

Spinal Muscular Atrophy (SMA) is an autosomal recessive neuromuscular disorder caused by homozygous deletions or mutations in the survival motor neuron 1 (*SMN1*) gene located at chromosome 5q13. With an estimated incidence of 1/6,000 to 1/10,000 live births (Pediatrician Branch of Chinese Medical Doctor Association et al., 2022), SMA represents the most prevalent fatal neurogenetic disorder in infancy and the primary genetic cause of mortality in children under 24 months of age (Pechmann and Kirschner, 2017). The disease pathogenesis involves progressive degeneration of *α*-motor neurons in the anterior horn of the spinal cord, leading to severe muscle atrophy and debilitating motor dysfunction. The therapeutic landscape has been transformed by disease-modifying treatments, particularly nusinersen – an antisense oligonucleotide that modulates SMN2 pre-mRNA splicing to enhance production of functional SMN protein. Clinical trials and real-world studies have consistently demonstrated significant improvements in motor milestones, survival rates, and respiratory function, with established safety profiles across pediatric populations (Wang et al., 2024; Vivo et al., 2019; Finkel et al., 2017; Yao et al., 2024; Wu et al., 2025).

Despite therapeutic advancements, longitudinal monitoring of treatment response remains problematic. Current evaluation protocols predominantly rely on standardized motor function scales, which present several limitations: (1) inherent subjectivity leading to inter-rater variability, (2) compromised assessment compliance due to developmental factors, cognitive limitations, and treatment-related fatigue in pediatric patients, and (3) significant rates of missing data during long-term follow-up (Yao et al., 2024; Xing et al., 2025). While promising biomarkers have been identified, including neurofilament light chain (NfL) and cerebrospinal fluid analytes, their clinical implementation faces substantial barriers. The invasive nature of sample collection, considerable costs, and poor tolerability in pediatric patients have hindered widespread adoption (Xing et al., 2025; Gao et al., 2025). Consequently, there is an urgent need to develop minimally invasive, cost-effective biomarkers suitable for serial monitoring in children. Serum-based biomarkers have emerged as particularly attractive candidates in this regard.

Among serum biomarkers, creatinine (Cr) has demonstrated correlation with motor function in SMA (Gao et al., 2025; Freigang et al., 2021; Alves et al., 2020), though its interpretation is confounded by renal function and nutritional status. The creatinine-to-cystatin C ratio (CCR), a readily available biochemical parameter, has been validated as a robust indicator of muscle mass in other neuromuscular and wasting disorders, with reduced susceptibility to confounding variables (Liu et al., 2024). More recently, the creatinine muscle index (CMI), a composite metric incorporating both Cr and cystatin C (CysC), has shown promise in providing enhanced accuracy for muscle mass estimation, demonstrating superior predictive performance compared to conventional measures in select populations (Ballew et al., 2023). While preliminary studies in adult SMA patients suggest CCR changes correlate with functional improvements during nusinersen treatment (Chen et al., 2025), these findings may not extrapolate to pediatric populations due to fundamental differences in growth dynamics, developmental physiology, and muscle metabolism. Notably, the utility of CCR and CMI as longitudinal biomarkers in pediatric SMA remains unvalidated, and their dynamic relationships with comprehensive motor function assessments have yet to be established.

To address these critical knowledge gaps, we designed a retrospective cohort study to systematically evaluate the longitudinal associations between serum biomarkers (Cr, CCR, CMI) and multidimensional motor function outcomes in pediatric SMA patients. This investigation aims to: (1) validate the clinical utility of these biomarkers for treatment monitoring, (2) characterize their dynamic relationships with functional measures, and (3) provide evidence-based recommendations for optimizing clinical assessment protocols and guiding future research directions in SMA management.

## Methods

### Study design, ethics, and patients

This single-center retrospective cohort study analyzed pediatric patients (age ≤18 years) with genetically confirmed 5q-spinal muscular atrophy (5q-SMA) who completed ≥18 months of standard intrathecal nusinersen treatment (12 mg/dose) between 1st December 2019 and 1st July 2025 at our tertiary care center. We obtained the case data through the hospital database from July 15 to August 31, 2025. Inclusion required homozygous deletion or compound heterozygous mutations in SMN1, while exclusion criteria comprised substantial missing data (>30% parameters) or loss to follow-up. The final cohort (*N* = 21) included SMA type 1 (9.5%, *n* = 2), type 2 (61.9%, *n* = 13), and type 3 (28.6%, *n* = 6), with documented demographics, SMN2 copy numbers, and baseline motor function.

The Institutional Review Board of Children’s Hospital of Chongqing Medical University approved this study, waiving informed consent per national regulations for retrospective analyses of anonymized clinical data. All procedures complied with the Declaration of Helsinki (2023), ensuring no therapeutic modifications were made for research purposes.

### Efficacy assessment and biomarker detection

This study evaluated treatment efficacy at the 18-month endpoint, a timepoint selected based on nusinersen’s well-characterized pharmacodynamic profile comprising an initial dose accumulation phase (0–6 months) followed by functional stabilization (6–18 months) (Chen et al., 2025; Acsadi et al., 2021). Motor function assessments were conducted by certified evaluators using standardized instruments: Hammersmith Functional Motor Scale-Expanded (HFMSE), Revised Upper Limb Module (RULM), Children’s Hospital of Philadelphia Infant Test of Neuromuscular Disorders (CHOP INTEND), and Hammersmith Infant Neurological Examination-2 (HINE-2) (Darras et al., 2019). Serial non-fasting venous samples collected at six study visits (V1-baseline through V8-18 months) underwent biochemical analysis including serum creatinine (Jaffe method) and cystatin C quantification. Derived parameters comprised the creatinine-to-cystatin C ratio (CCR) and creatinine muscle index (CMI), with CMI calculated as: [eGFRcys (mL/min/1.73 m^2^) × Serum Creatinine (mg/dL) × 1 dL/100 mL × 1,440 min/day], expressed in mg/day/1.73 m^2^ after body surface area normalization. Cystatin C-based estimated glomerular filtration rate (eGFRcys) was determined using the CAPA formula: 130 × Cystatin C^−1^·^069^ × Age^−0^·^117^–7 (Grubb et al., 2014), with all assays performed in our institution’s CLIA-certified core laboratory under standardized protocols.

### Statistical analysis

All statistical analyses were performed using Python 3.12 and R 4.5.1. Continuous variables were characterized as mean ± standard deviation for normally distributed data and median (interquartile range) for non-normally distributed data, while categorical variables were expressed as frequencies (percentages). Given the observed non-normal distribution of treatment-induced change values, the nonparametric Wilcoxon signed-rank test was systematically employed for all within-group longitudinal comparisons (each time point versus baseline), with Bonferroni correction applied to address multiple comparisons. This conservative approach ensured robust inference while maintaining methodological consistency across analyses.

**Figure 1:**
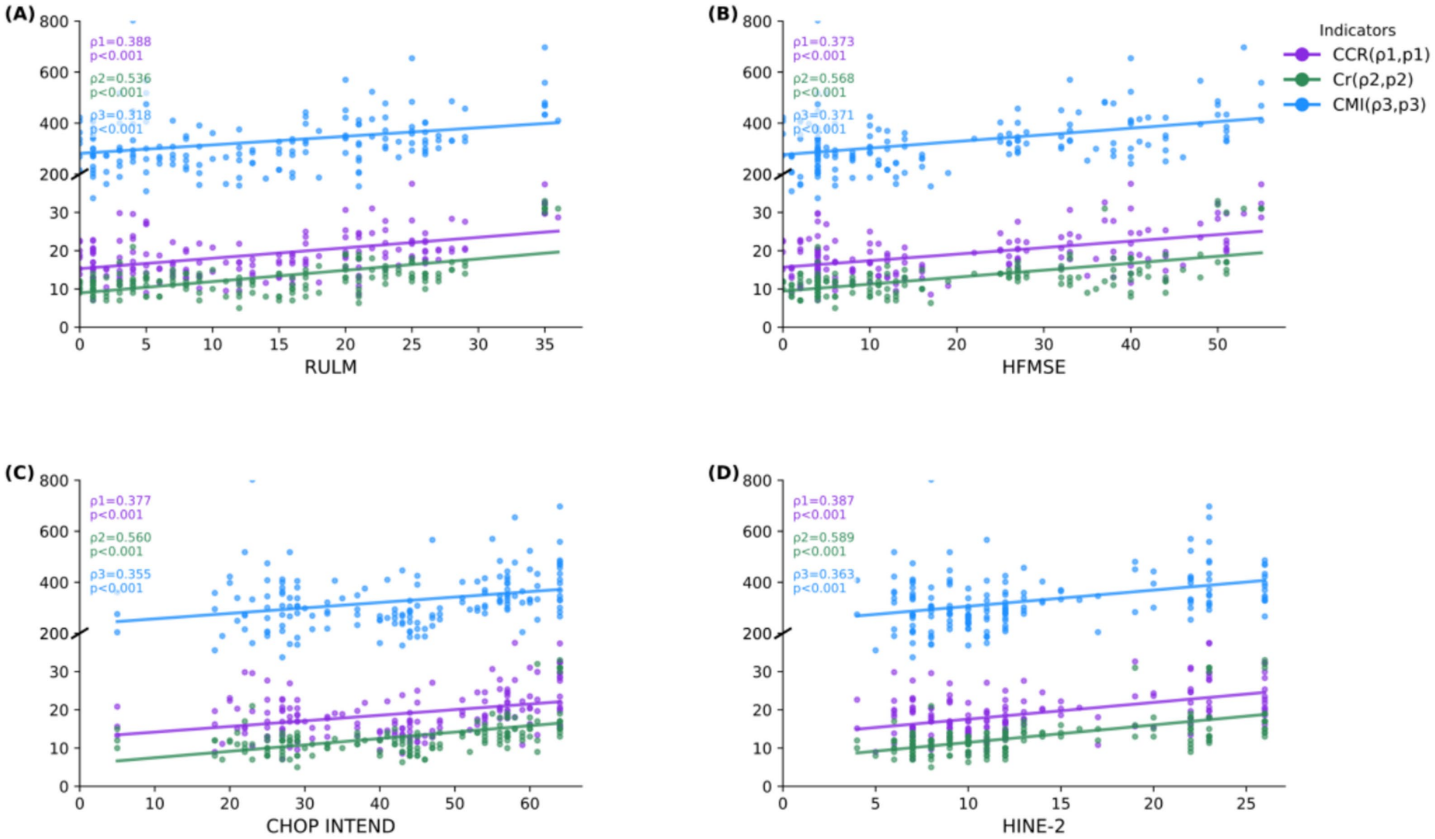
Correlations between functional data and laboratory parameters during nusinersen treatment. **(A)** Correlation between serum biomarkers and RULM score; **(B)** Correlation between serum biomarkers and HFMSE score; **(C)** Correlation between serum biomarkers and CHOP INTEND score; **(D)** Correlation between serum biomarkers and HINE-2 score. HFMSE: Hammersmith Functional Motor Scale Expanded; RULM: Revised Upper Limb Module; CHOP INTEND: Children’s Hospital of Philadelphia Infant Test of Neuromuscular Disorders; HINE-2: Hammersmith Infant Neurological Examination, Part 2; Cr: Serum Creatinine; CCR: Creatinine to Cystatin C Ratio; CMI: creatinine muscle index. The figure displays pairwise correlations and that axis orientation does not imply causality.

Baseline relationships between serum biomarkers and motor function were assessed using Spearman’s rank correlation analysis, with subsequent partial correlation analyses adjusting for potential confounders (age, SMA type, and albumin levels). For longitudinal evaluation, changes in biomarkers and functional scores over time were assessed using Wilcoxon signed-rank tests (each time point vs. baseline) (Table 1), and their associations were examined via Spearman’s rank correlation at each time point (Figure 1). Notably, subgroup analyses were not pursued due to sample size limitations that would compromise statistical power for meaningful interpretation.

**Table 1:** Comparison of serum biomarkers and functional assessments during treatment versus baseline.

Variable	Baseline data (V1)	2-month data (V4)	6-month data (V5)	10-month data (V6)	14-month data (V7)	18-month data (V8)
Cr (umol/l)	12.40 (11.14–15.00)	12.00 (11.00–13.00) *p* = 0.215	13.00 (10.00–14.00) *p* = 0.215	13.00 (10.00–14.00) *p* = 1.000	10.00 (8.00–14.00) *p* = 0.092	12.00 (9.00–13.00) *p* = 0.054
CCR	19.78 (16.67–23.08)	16.90 (12.82–20.00) *p* = 0.181	18.57 (14.49–21.88) *p* = 1.000	21.88 (17.02–25.00) *p* = 0.768	16.09 (13.43–19.67) *p* = 0.242	17.14 (12.90–19.23) *p* = 0.120
CMI	369.60 (284.94–409.90)	293.68 (241.30–338.80) *p* = 0.181	327.19 (259.82–371.61) *p* = 1.000	390.91 (288.62–450.62) *p* = 1.000	276.53 (236.49–345.75) *p* = 0.096	292.41 (207.79–327.56) ***p* = 0.016**
HFMSE	10.00 (4.00–33.00)	14.00 (4.00–32.00) *p* = 0.477	16.00 (4.00–33.00) ***p* = 0.030**	10.00 (4.00–37.00) ***p* = 0.024**	13.00 (6.00–36.00) ***p* = 0.004**	13.00 (4.00–33.00) *p* = 0.131
RULM	13.00 (4.00–20.00)	16.00 (4.00–22.00) *p* = 0.068	15.00 (4.00–22.00) *p* = 0.198	12.00 (3.00–22.00) *p* = 0.540	13.00 (6.00–23.00) *p* = 0.051	12.00 (5.00–21.00) *p* = 0.172
CHOP INTEND	38.00 (25.00–55.00)	45.00 (28.00–57.00) ***p* = 0.024**	45.00 (30.00–56.00) *p* = 0.095	45.00 (29.00–56.00) *p* = 0.173	46.00 (33.00–56.00) *p* = 0.056	44.00 (29.00–57.00) *p* = 0.484
HINE-2	10.00 (8.00–15.00)	11.00 (9.00–17.00) *p* = 0.771	11.00 (8.00–17.00) p = 1.000	11.00 (9.00–19.00) p = 1.000	12.00 (8.00–22.00) p = 1.000	11.00 (8.00–19.00) *p* = 0.769

HFMSE: Hammersmith Functional Motor Scale Expanded; RULM: Revised Upper Limb Module; CHOP INTEND: Children’s Hospital of Philadelphia Infant Test of Neuromuscular Disorders; HINE-2: Hammersmith Infant Neurological Examination, Part 2; Cr: Serum Creatinine; CCR: Creatinine to Cystatin C Ratio; CMI: Creatinine muscle index; All data were analyzed statistically using the Wilcoxon signed-rank test. p: *p*-value obtained after Bonferroni correction. Bolded *p*-values indicate *p* < 0.05.

*Wilcoxon test compares individual change directions, not group medians. A significant *p*-value indicates most patients improved, even if the median stayed the same.

A comprehensive missing data management protocol was implemented: creatinine values below the detection threshold (<13.3 μmol/L) were imputed via maximum likelihood estimation, while multiple imputation (m = 15) with predictive mean matching (PMM) was applied to motor function scores (HFMSE, RULM) and serum biomarkers. The imputation model incorporated clinically relevant covariates including patient ID (as categorical variable), visit timepoint, age, and SMA type, ensuring biologically plausible imputations. Excellent inter-rater reliability (ICC > 0.9) across functional assessments further validated data collection quality and consistency throughout the study.

## Results

### Participants

The final analytical cohort comprised 21 pediatric patients with 5q-SMA (type 1: *n* = 2 [9.5%]; type 2: *n* = 13 [61.9%]; type 3: *n* = 6 [28.6%]), with detailed baseline demographics and clinical characteristics summarized in Table 2. The study population had a mean age of 4.40 years (95% CI: 3.00–6.50) with male predominance (57.1%, *n* = 12), and 23.8% (*n* = 5) retained independent ambulation capacity at baseline. Pre-treatment (V1) motor function assessments revealed median Hammersmith Functional Motor Scale-Expanded (HFMSE) and Hammersmith Infant Neurological Examination-2 (HINE-2) scores of 10.00 (95% CI: 4.00–33.00 and 8.00–15.00, respectively), while Revised Upper Limb Module (RULM) and Children’s Hospital of Philadelphia Infant Test of Neuromuscular Disorders (CHOP INTEND) showed mean scores of 12.38 ± 9.82 and 40.05 ± 15.86, respectively. Baseline serum biomarker analysis demonstrated a right-skewed creatinine (Cr) distribution (12.40 μmol/L, 95% CI: 11.14–15.00), with cystatin C (Cys C) levels at 0.68 ± 0.15 mg/L, creatinine-to-cystatin C ratio (CCR) of 20.47 ± 5.21 μmol/mg, cystatin C-based estimated glomerular filtration rate (eGFRcys) of 166.36 ± 40.4 mL/min/1.73 m^2^, and creatinine muscle index (CMI) of 359.78 ± 95.56 (units as previously defined).

**Table 2:** Baseline characteristics.

Variable	Patients with SMA (*n* = 21)
Age (years)	4.40 (3.00–6.50)
Weight (kg)	15.00 (13.50–19.80)
Sex (male/female)	12/9
SMA type (1/2/3)	2/13/6
SMN2 copy number (≤3/>3)	20/1
Number = 4	1
Number = 3	19
Number = 2	1
Ambulant (yes/no)	5/16
HFMSE (score)	10.00 (4.00–33.00)
RULM (score)	12.38 ± 9.82
CHOP INTEND (score)	40.05 ± 15.86
HINE-2 (score)	10.00 (8.00–15.00)
Albumin (g/L)	46.82 ± 2.53
Cr (μmol/L)	12.40 (11.14–15.00)
Cystatin C (mg/L)	0.68 ± 0.15
CCR	20.47 ± 5.21
eGFRcys (mL/min/1.73m^2^)	166.36 ± 40.4
CMI	359.78 ± 95.56

HFMSE: Hammersmith Functional Motor Scale Expanded (score range 0–66); RULM: Revised Upper Limb Module (score range 0–37); CHOP INTEND: Children’s Hospital of Philadelphia Infant Test of Neuromuscular Disorders (score range 0–64); HINE-2: Hammersmith Infant Neurological Examination Part 2 (score range 0–26); Cr: serum creatinine (mol/L); CCR: creatinine to cystatin ratio; eGFRcys: Estimated glomerular filtration rate calculated using cystatin C, based on the CAPA formula (Asian pediatric population). CMI: creatinine muscle index, CMI = eGFRcys (mL/min per 1.73 m^2^) × serum creatinine (mg/dL) × 100 mL 1 dL × 1,440 min/day.

### Evolution of indices and functional scores during treatment

**Figure 2:**
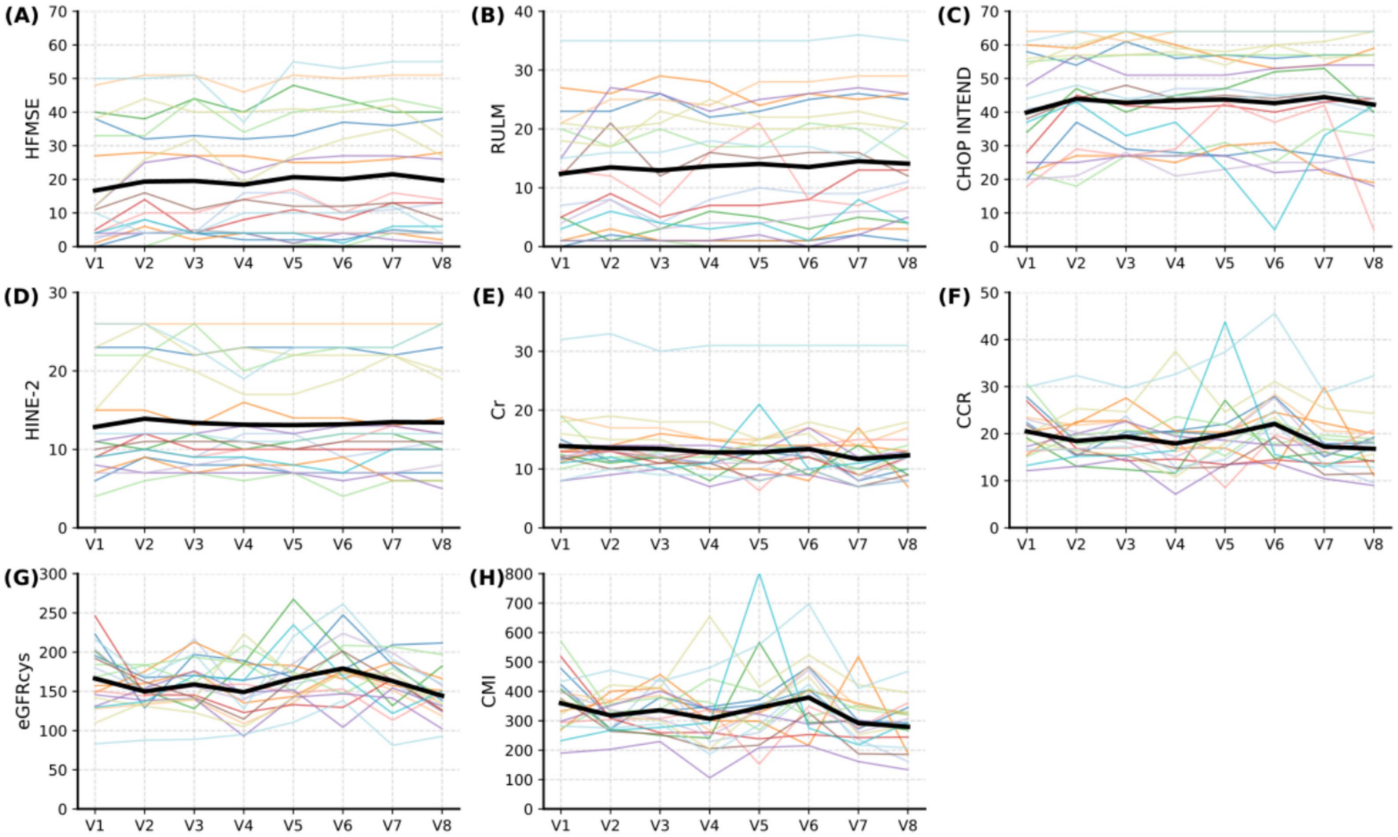
Changes in functional scores and serum biomarkers in SMA patients during nusinersen treatment. The thick black line represents the average change for all patients. HFMSE score, RULM score, CHOP INTEND score, and HINE-2 score showed an upward trend **(A–D)**, while Cr exhibited a slight downward trend **(E)**, and CCR, eGFRcys, and CMI demonstrated a fluctuating decline **(F–H)**. HFMSE: Hammersmith Functional Motor Scale Expanded (score range 0–66); RULM: Revised Upper Limb Module (score range 0–37); CHOP INTEND: Children’s Hospital of Philadelphia Infant Test of Neuromuscular Disorders (score range 0–64); HINE-2: Hammersmith Infant Neurological Examination Part 2 (score range 0–26); Cr: serum creatinine (μmol/L); CCR: creatinine to cystatin ratio; eGFRcys: Estimated glomerular filtration rate calculated using cystatin C; CMI: creatinine muscle index.

The study captured dynamic trajectories of functional outcomes and serum biomarkers throughout the treatment period (Figure 2), with comprehensive timepoint-specific comparisons detailed in Table 1. Motor function assessments collectively demonstrated progressive improvement, though with distinct temporal patterns: both Hammersmith Functional Motor Scale-Expanded (HFMSE) and Children’s Hospital of Philadelphia Infant Test of Neuromuscular Disorders CHOP INTEND) scores exhibited sustained incremental gains [At the 10-month assessment (V6), although the median HFMSE score remained unchanged from baseline, the significant *p*-value reflects consistent improvement in the majority of patients, as detected by the Wilcoxon signed-rank test], HFMSE showed significant improvements from baseline at months 6, 10, and 14 (*p* < 0.05), while CHOP INTEND reached significance only at month 2 (*p* = 0.024), after which improvements were not statistically significant (all *p* > 0.05). Revised Upper Limb Module (RULM) scores showed an initial improvement trend during the first 6–10 months; however, changes from baseline did not reach statistical significance at any timepoint (all *p* > 0.05). Hammersmith Infant Neurological Examination-2 (HINE-2) scores remained relatively stable throughout follow-up, with no significant differences detected at any visit compared to baseline (all *p* > 0.05, Table 1), despite a modest upward trend in median values (from 10.0 to 11.0–12.0). Serum creatinine (Cr) maintained remarkable stability throughout the observation window (Figure 2E), whereas creatinine-to-cystatin C ratio (CCR), cystatin C-based estimated glomerular filtration rate (eGFRcys), and creatinine muscle index (CMI) displayed more complex dynamics—including a transient CCR rebound between months 2–6 (V4-V6) and bimodal peaks for eGFRcys/CMI at month 6 (V6) preceding subsequent decline (Figures 2F–H).

Comparative analysis against baseline revealed differential treatment effects across assessment modalities. HFMSE achieved statistical significance at month 6 (V5; *p* = 0.030), month 10 (V6; *p* = 0.024), and month 14 (V7; *p* = 0.004), though this effect attenuated by month 18 (V8; *p* = 0.131). In contrast, RULM failed to reach significance at any timepoint (all *p* > 0.05) despite observable improvement trends. CHOP INTEND showed only transient month 2 (V4) enhancement (*p* = 0.024) despite median score elevation from 38 to 45 points, suggesting possible ceiling effects. Biomarker analysis confirmed group-level stability for Cr and CCR (all *p* > 0.05), while CMI demonstrated significant month 18 (V8) reduction (*p* = 0.016)—a finding requiring cautious interpretation due to potential formula-derived estimation biases and confounding metabolic variables.

Clinically meaningful improvement (defined as ≥3-point increase on HFMSE) was achieved in 28.57% of patients (*n* = 6/21), while 23.81% (*n* = 5/21) attained the RULM’s minimal clinically important difference (MCID; ≥2-point improvement). Longitudinal analysis demonstrated maintained creatinine (Cr) stability throughout the treatment period (all *p* > 0.05), while the creatinine-to-cystatin C ratio (CCR) exhibited a non-significant increasing trend at the 10-month timepoint (V6; *p* = 0.768 after Bonferroni correction). Further analysis revealed that eight patients (38.10%) exhibited stable or increasing CCR levels throughout treatment, which was associated with functional score improvements. In contrast, five patients (23.81%) showed a progressive decline in CCR, which was generally associated with a decrease in motor function.

### Adverse events

No treatment discontinuations occurred due to lumbar puncture failure. Among the cohort, 18 patients had scoliosis, one of whom underwent corrective spinal surgery during the treatment period; 1 additional case was suspected of osteochondroma. No serious adverse events related to intrathecal nusinersen administration were observed.

### Correlation between serum biomarkers and functional outcomes

Spearman correlation analysis at baseline (Table 3) demonstrated that CCR exhibited significant positive associations with multiple clinical parameters: ambulation ability (*ρ* = 0.609, *p* = 0.003), CHOP INTEND score (*ρ* = 0.458, *p* = 0.037), RULM score (*ρ* = 0.491, *p* = 0.024), HINE-2 score (*ρ* = 0.494, *p* = 0.023), HFMSE score (*ρ* = 0.470, *p* = 0.032), creatinine (Cr) levels (*ρ* = 0.730, *p* < 0.001), and SMA subtype classification (*ρ* = 0.503, *p* = 0.020). Conversely, CCR showed no statistically significant correlations with SMN2 copy number (*ρ* = 0.153, *p* = 0.508), serum albumin (*ρ* = −0.166, *p* = 0.618), or cystatin C levels (*ρ* = −0.294, *p* = 0.196).

**Table 3:** Correlation between baseline serum biomarker levels and motor function scores.

**Variable**	**Spearman**			**Partial**		
	**Cr (ρ, *p*-value)**	**CCR (ρ, *p*-value)**	**CMI (ρ, *p*-value)**	**Cr (ρ, *p*-value)**	**CCR (ρ, *p*-value)**	**CMI (ρ, *p*-value)**
Age (year)	−0.178, *p* = 0.441	−0.048, *p* = 0.836	−0.298, *p* = 0.189			
Sex (male/female)	0.000, *p* = 1.000	0.048, *p* = 0.837	0.016, *p* = 0.945			
SMA type	0.515, ***p* = 0.017**	0.503, ***p* = 0.020**	0.306, *p* = 0.177			
SMN2 copy number	0.166, *p* = 0.472	0.153, *p* = 0.508	0.051, *p* = 0.826	0.522, ***p* = 0.015**	0.164, *p* = 0.478	0.142, *p* = 0.540
Ambulant (yes/no)	0.722, ***p* < 0.001**	0.609, ***p* = 0.003**	0.462, ***p* = 0.035**			
HFMSE	0.711, ***p* < 0.001**	0.470, ***p* = 0.032**	0.388, *p* = 0.082	0.626, ***p* = 0.002**	0.369, *p* = 0.100	0.405, *p* = 0.068
RULM	0.740, ***p* < 0.001**	0.491, ***p* = 0.024**	0.352, *p* = 0.117	0.684, ***p* = 0.001**	0.329, *p* = 0.146	0.342, *p* = 0.130
CHOP INTEND	0.724, ***p* < 0.001**	0.458, ***p* = 0.037**	0.320, *p* = 0.157	0.643, ***p* = 0.002**	0.218, *p* = 0.342	0.256, *p* = 0.263
HINE-2	0.780, ***p* < 0.001**	0.494, ***p* = 0.023**	0.396, *p* = 0.076	**–**	–	–
Albumin (g/L)	0.225, *p* = 0.326	−0.116, *p* = 0.618	−0.081, *p* = 0.729			
Cr (μmol/L)		0.730, ***p* < 0.001**	0.686, ***p* = 0.001**		**–**	
Cystatin C (mg/L)	0.355, *p* = 0.115	−0.294, *p* = 0.196	−0.335, *p* = 0.138	–0.487, ***p* = 0.025**	−0.312, *p* = 0.169	−0.329, *p* = 0.146
CCR	0.730, ***p* < 0.001**		0.944, ***p* < 0.001**	0.466, ***p* = 0.033**		0.981, *p* < 0.001
CMI	0.686, ***p* = 0.001**	0.944, *p* < 0.001		0.465, ***p* = 0.034**	0.981, *p* < 0.001	

HFMSE: Hammersmith Functional Motor Scale Expanded; RULM: Revised Upper Limb Module; CHOP INTEND: Children’s Hospital of Philadelphia Infant Test of Neuromuscular Disorders; HINE-2: Hammersmith Infant Neurological Examination, Part 2; Cr: Serum Creatinine; CCR: Creatinine to Cystatin C Ratio; CMI: creatinine muscle index; Partial correlation analysis was adjusted for the effects of age, SMA subtype, and serum albumin level. Due to the limited sample size and the presence of multicollinearity, valid estimates could not be obtained for certain variables in the partial correlation analysis after adjusting for confounding factors such as age, SMA subtype, and albumin level. Bold font indicates *p* < 0.05.

CMI displayed significant positive correlations with ambulation capacity (*ρ* = 0.462, *p* = 0.035) and demonstrated particularly strong associations with both Cr (*ρ* = 0.686, *p* = 0.001) and CCR (*ρ* = 0.944, *p* < 0.001). However, CMI failed to show statistically significant relationships with any motor function assessments—HFMSE (*ρ* = 0.388), RULM (*ρ* = 0.352), CHOP INTEND (*ρ* = 0.320), and HINE-2 (*ρ* = 0.396)—as all corresponding *p*-values exceeded the 0.05 significance threshold.

Cr concentrations showed robust positive correlations with all motor function metrics: HFMSE (*ρ* = 0.711), RULM (*ρ* = 0.740), CHOP INTEND (*ρ* = 0.724), and HINE-2 (*ρ* = 0.780; all *p* < 0.001). Additionally, Cr demonstrated significant associations with ambulation ability (*ρ* = 0.722, *p* < 0.001), CCR (*ρ* = 0.730, *p* < 0.001), CMI (*ρ* = 0.686, *p* = 0.001), and SMA subtype classification (*ρ* = 0.515, *p* = 0.017). No significant correlation was observed between Cr levels and SMN2 copy number (*ρ* = 0.166, *p* = 0.472).

After controlling for age, SMA subtype, and albumin levels through partial correlation analysis, Cr maintained strong, statistically significant associations with motor function scores (HFMSE, RULM, and CHOP INTEND; *ρ* = 0.626–0.684, all *p* ≤ 0.002), CCR (*ρ* = 0.465, *p* < 0.035), and CMI (*ρ* = 0.466, *p* < 0.035), indicating these relationships were independent of these potential confounders. In contrast, the adjusted correlations between CCR and motor function scores were attenuated and no longer significant (e.g., HFMSE: *ρ* = 0.369, *p* = 0.100). Similarly, CMI showed no significant correlations with any motor function measures after adjustment (HFMSE: *ρ* = 0.405, *p* = 0.068; RULM: *ρ* = 0.342, *p* = 0.130; CHOP INTEND: *ρ* = 0.256, *p* = 0.263), suggesting these associations may be mediated by the controlled clinical factors.

Analysis of dynamic correlations during treatment revealed moderate but significant associations between both CCR and CMI with motor function scores (Figures 1A–D). CCR showed positive correlations with RULM (*ρ* = 0.373), HFMSE (*ρ* = 0.568), CHOP INTEND (*ρ* = 0.458), and HINE-2 (*ρ* = 0.492; all *p* < 0.001). Similarly, CMI demonstrated significant, though more modest, correlations across all functional measures (*ρ* = 0.318–0.371; all *p* < 0.001). Notably, these association strengths were consistently weaker than those observed for Cr, which maintained stronger correlations with all motor function scores (*ρ* = 0.536–0.589; all *p* < 0.001) throughout the treatment period.

## Discussion

This pioneering study provides the first comprehensive longitudinal evaluation Cr, CCR, and CMI dynamics during nusinersen therapy in pediatric SMA, systematically investigating their temporal relationships with multidimensional motor function assessments. Key discoveries reveal: (1) Significant motor function improvement under nusinersen treatment, particularly evidenced by HFMSE progression; (2) Serum Cr exhibited robust, clinically relevant correlations with all motor function scores that remained statistically significant after adjustment for age, SMA subtype, and albumin levels, demonstrating consistent performance across varying analytical assumptions; (3) While baseline CCR showed moderate functional correlations, its longitudinal associations were attenuated after confounding adjustment and exhibited greater sensitivity to missing data handling methods. While prior adult SMA studies noted Cr-function correlations (Freigang et al., 2021; Alves et al., 2020), this work uniquely confirms its longitudinal reliability in children, where preserved renal function minimizes a major confounding variable (Groothof et al., 2024). Cr’s well-documented relationship with skeletal muscle mass, disease severity, and functional status (Uzan et al., 2022) positions it as an objective complement to motor scales, particularly where standardized assessments face implementation barriers.

Contrary to adult findings (Chen et al., 2025), neither CCR nor CMI demonstrated superior performance to Cr alone in this cohort, highlighting age-dependent biomarker dynamics. While CMI correlated with ambulation (*ρ* > 0.68) and tracked Cr/CCR, it showed no significant motor function associations and exhibited paradoxical longitudinal decline (V8: *p* = 0.016)—likely reflecting methodological instability from its eGFRcys-dependent calculation. The CAPA equation’s inherent bias (Kuang et al., 2022) and children’s preserved renal function amplify estimation errors during growth. Similarly, CCR’s baseline functional correlations (*ρ* = 0.458–0.609) became nonsignificant after adjustment for age/subtype/albumin, with sensitivity analyses revealing instability under missing-data assumptions. Pediatric physiology fundamentally alters biomarker interpretation: normally low Cr levels (exacerbated by SMA-related atrophy), high growth-related metabolic activity, and Cys C’s intrinsic variability (age/weight/inflammation effects; Knight et al., 2004; Li et al., 2012) render CCR’s renal “correction” counterproductive. Age-adjusted Cr emerges as the most robust pediatric marker, directly reflecting muscle deficit against developmental expectations (Liu et al., 2019; Liu et al., 2019). These findings caution against adult-derived biomarker extrapolations, emphasizing the need for age-specific algorithms in pediatric neuromuscular care.

The interpretation of functional outcomes necessitates a multidimensional analytical approach. While HFMSE scores demonstrated robust treatment effects across both primary and sensitivity analyses (varying missing-data imputations), establishing their reliability as a primary efficacy endpoint (Coratti et al., 2025), RULM and CHOP INTEND exhibited greater methodological sensitivity—particularly under pessimistic missing-data assumptions where treatment-related improvements at critical timepoints lost statistical significance. This analytical fragility complicates the attribution of subtle functional changes to therapeutic intervention, particularly for upper limb (RULM) and infant-specific (CHOP INTEND) outcomes, underscoring the need for larger confirmatory trials with standardized assessment protocols. These limitations highlight the clinical imperative to integrate objective biomarkers like Cr, which demonstrated consistent correlations with motor function and stability across analytical models, as complementary outcome measures to enhance the reliability of therapeutic monitoring in pediatric SMA.

Our study revealed a clinically significant dissociation between functional and biomarker trajectories during nusinersen treatment: while motor function demonstrated progressive improvement, Cr, CCR, and CMI exhibited concurrent declines. This paradoxical pattern reflects nusinersen’s proposed mechanism of action (Finkel et al., 2017; Hoy, 2017)—enhancing neuromuscular junction transmission efficiency and motor neuron survival without necessarily restoring muscle bulk, thereby enabling improved functional performance despite persistent (though potentially attenuated) muscle atrophy. The observed biomarker dynamics suggest these parameters may serve as sensitive indicators of treatment-induced metabolic adaptation, with declining Cr/CCR/CMI values potentially signaling reduced muscle catabolic demand rather than pure mass loss. Importantly, this dissociation underscores the complementary roles of functional scales (capturing clinical efficacy) and muscle biomarkers (reflecting underlying biological response) in comprehensive therapeutic monitoring, particularly for assessing treatment durability beyond initial functional gains.

This investigation acknowledges several methodological constraints inherent in its design. The cohort’s heterogeneity—spanning diverse ages and SMA subtypes—necessitated divergent functional assessments (CHOP INTEND/HINE-2 for type 1 infants vs. HFMSE/RULM for types 2–3) (Yang et al., 2020), potentially introducing scale-dependent confounding in biomarker-function correlations. With limited subgroup representation (*n* = 2 type 1), our reported associations between Cr and motor outcomes reflect pooled trends across this phenotypically mixed pediatric population rather than subtype-specific relationships. While these robust correlations suggest Cr’s broad utility as a monitoring biomarker, their generalizability to discrete clinical subgroups—particularly in type 1 SMA or distinct developmental stages—requires validation through larger, phenotype-stratified studies with standardized assessment protocols.

## Limitations

This single-center retrospective study is limited by sample size constraints and potential selection bias, despite employing multiple imputation and sensitivity analyses to address missing data. The absence of an untreated control group limits our ability to fully isolate treatment effects from natural developmental trajectories. However, with nusinersen now established as the standard of care, withholding treatment to create a concurrent control arm is ethically untenable. Given that the natural history of SMA is defined by progressive functional decline, the within-subject improvements observed in our cohort provide compelling, albeit indirect, evidence of treatment-associated benefit. We acknowledge that this design does not offer the causal certainty of a randomized controlled trial. We hope that future studies—particularly as therapies such as risdiplam become more widely accessible and reimbursed—may incorporate active comparator arms or prospectively enrolled cohorts with staggered treatment initiation to enable more definitive causal inference. The exclusion of emerging biomarkers like neurofilament light chain (NfL) restricts mechanistic insights. Future multicenter prospective studies integrating Cr with disease-specific markers to develop comprehensive models assessing neuromuscular function, muscle mass dynamics, and neuroaxonal integrity concurrently in pediatric SMA.

## Conclusion

Our findings support Cr as a reliable, robust, and practical dynamic biomarker for tracking treatment response to nusinersen in pediatric SMA. In contrast, CCR and the CMI showed limited suitability for long-term monitoring in this pediatric population, largely due to confounding by growth-related factors. These findings should be validated in larger prospective pediatric cohorts, and further efforts are needed to establish age-specific reference ranges.

## Data Availability

The original contributions presented in the study are included in the article/supplementary material, further inquiries can be directed to the corresponding author/s.
